# Antibiotic residues in meat and feed in Kazakhstan: A nationwide surveillance study on food safety and antimicrobial resistance risks

**DOI:** 10.14202/vetworld.2025.2839-2849

**Published:** 2025-09-23

**Authors:** Akanova Zhannara, Assauova Zhenisgul, Uskenov Rashit, Suranshiyev Zhanbolat, Sharipova Galina, Shaikenova Kymbat, Akibekov Orken

**Affiliations:** 1 Joint Kazakh-Chinese Laboratory for Biological Safety, Kazakh Agro-Technical Research University, Astana, Kazakhstan; 2 Department of Veterinary Sanitation, Kazakh Agro-Technical Research University, Astana, Kazakhstan; 3 Department of Production and Processing of Animal Products, Kazakh Agro-Technical Research University, Astana, Kazakhstan; 4 Department of Microbiology and Biotechnology, Kazakh Agro-Technical Research University, Astana, Kazakhstan

**Keywords:** antibiotics, antimicrobial resistance, feed, food safety, Kazakhstan, meat, residues

## Abstract

**Background and Aim:**

The widespread use of antibiotics in livestock production enhances growth and prevents disease but contributes to antimicrobial resistance (AMR) and food contamination through residual accumulation in animal-derived products. Limited national-level data exist for Kazakhstan, where livestock farming is a major agricultural sector. This study aimed to evaluate antibiotic residues in meat and feed samples collected from across Kazakhstan and assess their implications for public health and food safety.

**Materials and Methods:**

A cross-sectional survey was conducted between December 2023 and March 2025 across 14 regions of Kazakhstan. A total of 1,026 meat samples (beef, horse, chicken, lamb, and pork) and 150 feed samples (succulent, coarse, concentrated) were collected from licensed facilities. Samples were processed under standard protocols and analyzed using the Evidence Investigator biochip system (Randox, UK), employing Antimicrobial Array I Ultra and Antimicrobial Array II Plus panels. Statistical analyses, including analysis of variance, were performed using International Business Machine Statistical Package for the Social Sciences v25, with statistical significance set at p < 0.05.

**Results:**

Residual antibiotics were detected in all categories of meat and feed, with several concentrations exceeding permissible limits. Succulent feeds showed the highest contamination (streptomycin 86.43 ppb; quinolones 35.56 ppb). Among meats, chicken contained the highest residue levels (quinolones up to 91.97 ppb; streptomycin up to 492.00 ppb), followed by beef (sulfadimethoxine 18.26 ppb; dapsone up to 285.14 ppb). Statistically significant differences were observed among meat types for quinolones (p = 0.000), ceftiofur (p = 0.000), thiamphenicol (p = 0.003), tylosin (p = 0.000), and tetracyclines (p = 0.005). Streptomycin levels varied widely but were not statistically significant (p = 0.072).

**Conclusion:**

The findings highlight uncontrolled antibiotic use in Kazakhstan’s livestock sector, particularly in poultry farming. The presence of elevated antibiotic residues in meat and feed underscores urgent food safety concerns and the potential acceleration of AMR. Strengthened regulatory oversight, strict adherence to drug withdrawal periods, and adoption of sustainable alternatives such as probiotics and phytogenic feed additives are crucial. Establishing a national monitoring program and expanding laboratory surveillance capacity are essential steps to safeguard public health and promote safe, sustainable livestock production.

## INTRODUCTION

In industrial livestock farming, antibiotics are extensively used to promote growth and prevent disease. However, their unregulated use has resulted in significant threats to human health and environmental safety [1]. Residual antibiotics in animal feed and meat contribute to the proliferation of antibiotic-resistant bacteria, elevating the risk of challenging infections [2]. The World Health Organization identifies antimicrobial resistance (AMR) as one of the greatest threats to global health, predicting that by 2050, deaths from resistant infections could exceed those caused by cancer [3]. The agricultural use of antibiotics began in the 1940s, following the discovery that subtherapeutic doses enhanced animal growth [4]. In 1951, the United States Food and Drug Administration formally approved the addition of antibiotics to animal feed without requiring veterinary prescriptions [5], although the 1969 Swann Report recommended restricting the use of medically important antibiotics as growth promoters [6].

A recent study by Åkerfeldt *et al*. [7] confirms that antibiotics used in livestock persist in meat products even after cooking and may adversely impact human health. These compounds also enter the environment through livestock waste, contaminating soil and water sources, and promoting the spread of resistant bacterial strains [3, 8]. For instance, a study by Yang *et al*. [6] in China found that 84% of environmental antibiotic emissions were attributable to livestock production. Multiple classes of antimicrobial compounds have been detected in surface and groundwater near pig and poultry farms, suggesting that the use of livestock waste as fertilizer introduces antimicrobial residues into the environment [9]. Despite the risks associated with pathogen resistance, subtherapeutic antibiotic use remains common in animal husbandry [10]. Tetracyclines, among the most widely used antibiotics in veterinary medicine, are frequently detected as residues in animal-derived products and in the environment [11]. Their widespread use in subtherapeutic doses is closely linked to increasing resistance among pathogenic microflora [12].

Regulatory responses have varied worldwide. In 2006, the European Union banned the use of antibiotics as growth promoters, while the United States implemented stricter regulations after 2013 [13]. In many developing countries, however, limited oversight has led to high residue levels in meat products. Farmers’ lack of awareness regarding the risks of antibiotic use often results in excessive and improper application [14]. Within the Eurasian Economic Union (EAEU), the Technical Regulation (TR) “On the Safety of Poultry Meat and Its Processed Products” (TR EAEU 051/2021) establishes hygiene standards, including maximum allowable limits for antibiotic residues [15]. Additional relevant regulations include the Law of the Republic of Kazakhstan “On Veterinary Medicine,” the EAEU TR “On Food Safety” (TR TS 021/2011), and sanitary and hygienic standards under the Customs Union. According to TR EAEU 051/2021, poultry meat must not contain chloramphenicol (<0.0003 mg/kg), tetracyclines (<0.01 mg/kg), or bacitracin (<0.02 mg/kg). These thresholds are designed to regulate antibiotic use in livestock and prevent harmful accumulation in the human body through consumption. Compliance is monitored by veterinary and sanitary authorities using advanced techniques such as high-performance liquid chromatography and mass spectrometry.

Kazakhstan currently lacks official statistical data on antibiotic use in the livestock and poultry sectors. Large agribusinesses often monitor usage, but proper dosage and compliance with veterinary prescriptions are not always ensured. In contrast, small private farms frequently lack adequate knowledge, resulting in uncontrolled application and adverse outcomes [16]. Safeguarding the health and welfare of food-producing animals is both ethically important and essential for ensuring food safety. Although antibiotics remain vital in veterinary care, their usage is increasingly scrutinized in light of the global surge in AMR [17].

One potential solution is organic livestock farming, which eliminates the use of antibiotics and synthetic additives. Organic meat generally contains fewer antibiotic residues, making it a safer option for consumers [2]. Transitioning to organic production not only lowers the risk of antibiotic resistance but also reduces environmental harm [4].

Despite increasing global concern about AMR, there is a lack of comprehensive national-level data on residual antibiotic concentrations in meat and feed in Kazakhstan. Most available studies are limited to specific regions, small sample sizes, or individual antibiotic classes, making it difficult to capture the true scale of contamination across the livestock production chain. Furthermore, while international regulations such as those of the European Union and the United States have led to significant reductions in antibiotic residues, similar large-scale surveillance programs have not been fully implemented in Kazakhstan. Regulatory frameworks, such as TR EAEU 051/2021, define permissible limits for poultry meat, but there is little evidence on compliance levels, enforcement, or how different livestock sectors contribute to antibiotic contamination. In addition, while previous research has primarily focused on meat products, the role of livestock feed as a potential reservoir and transmission route of antibiotic residues has received limited attention in the Kazakh context. This gap is critical because contaminated feed can directly influence residue accumulation in animal tissues and ultimately enter the food chain. Collectively, these limitations highlight an urgent need for systematic, nationwide surveillance of antibiotic residues across multiple livestock products and feed sources to inform evidence-based policy and strengthen food safety standards.

**Figure 1:**
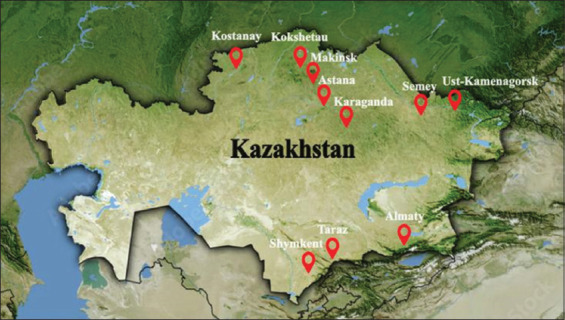
Geographical points of meat sampling in Kazakhstan [Source: The map was generated using QGIS (Desktop 3.x)].

This study aimed to conduct a nationwide assessment of residual antibiotic levels in both meat and animal feed across 14 regions of Kazakhstan (Table 1 and Figure 1). Specifically, it sought to (i) quantify antibiotic residues in commonly consumed meat types (beef, horse, chicken, lamb, and pork) and different feed categories (succulent, coarse, and concentrated), (ii) evaluate whether residue concentrations exceed internationally and regionally permissible limits, (iii) identify species-specific and feed-type-specific variations in contamination levels, and (iv) assess the potential implications for food safety and public health. By establishing a robust dataset and providing statistically validated evidence, this study intends to fill the existing knowledge gap, support the development of a national surveillance system, and contribute to the formulation of regulatory measures and alternative farming strategies that reduce antibiotic dependence in Kazakhstan’s livestock sector.

**Table 1:** Cartographic data.

No.	City	Country	Latitude	Longitude
1	Karaganda	Kazakhstan	49.8047	73.1022
2	Astana	Kazakhstan	51.1605	71.4704
3	Almaty	Kazakhstan	43.2389	76.8897
4	Kokshetau	Kazakhstan	53.2833	69.3833
5	Ust-Kamenagorsk	Kazakhstan	49.9714	82.6111
6	Makinsk	Kazakhstan	52.6333	70.4167
7	Kostanay	Kazakhstan	53.2144	63.6246
8	Semey	Kazakhstan	50.4111	80.2275
9	Taraz	Kazakhstan	42.9	71.3667
10	Shymkent	Kazakhstan	42.3167	69.595

## MATERIALS AND METHODS

### Ethical approval

The study did not require ethical approval because it was based on the analysis of commercially available food and feed samples and did not involve experiments on live animals.

### Study period and location

This cross-sectional study was conducted from December 2023 to March 2025 across 14 regions of Kazakhstan, targeting the largest meat production centers.

### Sampling strategy

Sampling locations were chosen based on livestock density and meat production volume. Major cities, such as Almaty, Astana, Shymkent, and Karaganda, known for their intensive meat processing and livestock production (including cattle, poultry, and horses), were included in the sampling plan.

### Sample collection and processing

#### Meat sampling

A total of 1,026 meat samples were collected comprising 486 in 2023 and 540 in 2024. Samples included beef, horse meat, chicken, lamb, and pork. They were obtained from licensed slaughterhouses, meat processing plants, cold storage units, and official storage facilities. All collection sites operated under veterinary supervision and held appropriate certification and permits. Each sample was placed in a sterile plastic bag, labeled with a unique code, stored at ≤4°C, and delivered to the laboratory within 24 h, maintaining the cold chain.

#### Feed sampling

Between 2023 and 2024, 150 feed samples were collected, including succulent, coarse, and concentrated types, along with domestic and imported feed additives. Feed sampling was conducted concurrently with meat collection in the same regions. Standard procedures were followed, as outlined in government standard (GOST) 27262–87 for vegetable feed and GOST 13586.3–83 for grain and feed additives [18, 19]. A minimum of 10-point samples per batch were collected using a stainless-steel probe. Subsamples were combined into a composite sample, labeled, and sealed in dry plastic bags. Hay and straw samples were transported at room temperature within 2–3 days, while silage and haylage were transported chilled within 24 h.

### Sample preparation

All samples were dried in a cabinet oven at 60°C for 12 h in the laboratory. The dried material was ground using an LM-202 mill and sieved through a C20/50 mesh (1 mm). For antibiotic extraction, 1 g of crushed feed was mixed with water (7:3) and 5 mL of methanol, vortexed for 1 min, and centrifuged at 2,880 × *g* for 10 min at 25^o^C. A 125 μL aliquot of the supernatant was collected. The residue was treated with 5 mL of extraction buffer (Cat. No. EV3724 Randox Laboratories Ltd., Crumlin, UK), mixed, and centrifuged under the same conditions, after which another 125 μL aliquot was collected and pooled with the first. The combined 250 μL extract was diluted with 750 μL of washing buffer (1:40 dilution) for analysis. All samples were cataloged in the laboratory logbook and assigned individual codes. Preliminary tests for moisture content and sample homogeneity were not performed.

### Determination of antibiotic residues

#### Analytical equipment and reagents

Antibiotic residue levels were determined using the Evidence Investigator biochip system (Randox, UK). Two validated analytical panels were employed:

- Antimicrobial Array I Ultra (Cat. No. EV3843)
- Antimicrobial Array II Plus (Cat. No. EV4169/A).

The assay is based on a solid-phase chemiluminescent enzyme immunoassay enzyme-linked immunosorbent assay-X. In this method, antibiotics in the sample compete with immobilized antigens for binding to horseradish peroxidase-labeled antibodies on a microchip. After washing, a substrate is added, and the resulting luminescence is measured. The signal intensity is inversely proportional to antibiotic concentration.

Calibration was performed using a 9-point standard curve. Calibrators were reconstituted in 1 mL of deionized water, vortexed for 30 min, and immediately used. Each kit also included quality control materials for validation of performance. All procedures followed Randox validation protocols and the guidelines of the European Medicines Agency.

### Data processing and statistical analysis

Statistical analyses were conducted using the International Business Machine Statistical Package for the Social Sciences Statistics version 25.0 (IBM Corp., Armonk, NY, USA). Descriptive statistics (mean, standard deviation, minimum, maximum) were calculated. The Shapiro-Wilk test was applied to assess the normality of data distribution. Group comparisons were performed using analysis of variance, with statistical significance set at p < 0.05.

## RESULTS

### Overview of sample analysis

Various meat and feed samples collected from 14 cities across Kazakhstan were analyzed. Two critical analytical parameters were considered: The limit of detection (LOD) (Table 2) and the maximum residue limit (MRL). The LOD indicates the lowest concentration of a substance that can be reliably detected by the method, reflecting equipment sensitivity. In contrast, MRLs represent the legally established safe limits of antibiotic residues permissible in food products for human consumption. Importantly, concentrations above the LOD may still remain within safe consumption limits if below the MRL.

**Table 2:** Limit of detection of antibiotics in feed.

Detection limit	Types of antibiotics					

	Quinolones	Ceftiofur	Thiamphenicol	Streptomycin	Tylosin	Tetracycline
Detection limit (parts per billion)	10	15	15	80	10	10

### Antibiotic residues in feed samples

The Randox biochip system was used to detect antibiotic residues in feed, enabling the identification of minimal concentrations critical to food safety risk assessment. Table 3 presents the statistical analysis of antibiotic residues across succulent, coarse, and concentrated feeds.

**Table 3:** Antibiotic content in various feed types.

Antibiotic	n	+	Average, ppb	δ	min	max	p-value
CEF							
Succulent	26	19	32.23 ± 4.93*	21.48	2.53	87.13	0.17
Roughage	31	21	29.28 ± 4.30	19.68	1.45	68.94	
Concentrated	78	20	18.52 ± 6.51	29.13	1.63	133.12	
QNL							
Succulent	26	20	35.56 ± 15.30	68.44	1.40	316.00	0.368
Roughage	31	22	11.92 ± 1.51	7.09	2.07	28.12	
Concentrated	78	23	20.14 ± 13.55	64.98	1.17	316.00	
TAF							
Succulent	26	19	8.62 ± 1.19	5.18	0.55	18.83	0.002
Roughage	31	22	6.35 ± 0.88	4.12	0.63	14.73	
Concentrated	78	23	3.66 ± 0.71	3.39	0.40	16.02	
STR							
Succulent	26	19	86.43 ± 52.03	226.77	1.82	984.00	0.402
Roughage	31	18	41.90 ± 21.43	90.93	0.00	374.42	
Concentrated	78	18	24.25 ± 3.38	14.32	1.06	48.14	
TIL							
Succulent	26	19	4.96 ± 0.52	2.28	0.90	8.46	0.006
Roughage	31	22	4.46 ± 0.57	2.66	1.08	12.83	
Concentrated	78	23	2.70 ± 0.42	2.03	0.40	8.62	
TCN							
Succulent	26	19	17.56 ± 1.74	7.60	6.78	36.64	0.02
Roughage	31	18	12.73 ± 1.24	5.25	0.00	21.45	
Concentrated	78	20	11.56 ± 1.60	7.14	3.76	36.14	

* Mean ± standard error, N = Number of samples, min = Minimum, max = Maximum, δ = Standard deviation

- Succulent feeds: Antibiotic concentrations significantly exceeded LODs, with streptomycin (86.43 ppb), quinolones (35.56 ppb), and ceftiofur (CEFT) (32.23 ppb) being most prominent. These elevated values may be linked to storage and fermentation processes.
- Coarse feeds: Residues were lower than in succulent feeds, but CEFT (29.28 ppb) and quinolones (11.92 ppb) still surpassed LODs.
- Concentrated feeds: These exhibited the lowest contamination levels, though none were entirely free of residues. Notable values included streptomycin (24.25 ppb) and quinolones (20.14 ppb).

Overall, antibiotic concentrations in feed exceeded LODs, particularly for quinolones and CEFT, highlighting potential public health risks. The greatest exceedances were observed in succulent feeds, where quinolone and CEFT levels surpassed LODs by 255.6% and 114.9%, respectively.

### Antibiotic residues in meat samples

**Figure 2:**
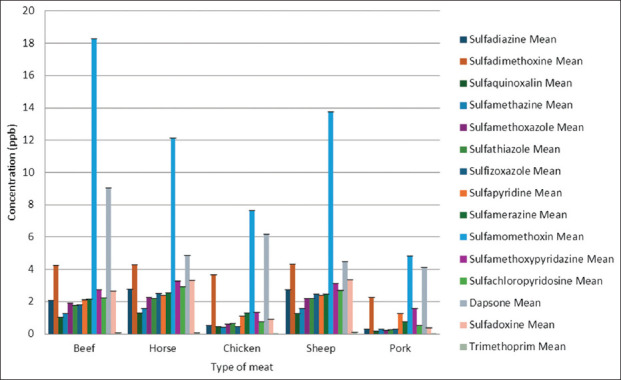
Residual antibiotic concentrations in different meat types.

In addition to feed, antibiotic residues were evaluated in chicken, beef, and lamb samples, focusing on 15 commonly used sulfonamide antibiotics. Results are summarized in Figure 2 and Appendix A.

#### Sulfonamides and trimethoprim

Residue analysis revealed notable interspecies differences. Beef showed the highest average concentrations of sulfamonomethoxine (18.26 ± 5.89 ppb) and dapsone (9.04 ± 2.01 ppb), with extreme values reaching 1125.00 and 285.14 ppb, respectively. Chicken and pork generally exhibited the lowest levels; sulfadiazine, sulfaquinoxaline, and sulfachloropyridazine rarely exceeded 1 ppb. Horse meat and mutton typically showed levels comparable to beef. The most significant interspecies differences were observed for sulfamethoxazole, sulfamethazine, sulfamerazine, and sulfadoxine (p < 0.001). Trimethoprim showed minimal residue levels (≤0.11 ppb). These findings highlight the need for species-specific monitoring standards.

#### Other antibiotic groups

Using the Antimicrobial Array II Plus, residue levels were determined for six major groups: quinolones, CEFT, thiamphenicol, streptomycin, tylosin, and tetracycline (Table 4).

**Table 4:** Residual amount of antibiotics in meat detected using the antimicrobial array II plus.

Antibiotic	Type of meat	n	Average, ppb	δ	min	max	p-value
Quinolones	Beef meat	171	0.82 ± 0.07	0.92306	0.00	3.83	0.000
	Sheep meat	27	1.10 ± 0.22	1.14599	0.00	5.20	
	Horse meat	63	0.80 ± 0.11	0.84851	0.00	3.58	
	Chicken	61	6.53 ± 2.03	15.82123	0.00	91.97	
	Pork	29	1.26 ± 0.19	1.00338	0.00	3.08	
Ceftiofur	Beef meat	171	2.82 ± 0.29	3.84622	0,00	17.41	0.000
	Sheep meat	27	0.86 ± 0.32	1.65925	0.00	8.02	
	Horse meat	63	1.02 ± 0.20	1.58041	0.00	5.81	
	Chicken	61	3.62 ± 0.63	4.88973	0.00	18.69	
	Pork	29	3.34 ± 0.92	4.95991	0.00	13.45	
Thiamphenicol	Beef meat	171	1.58 ± 0.11	1.43815	0.00	5.60	0.003
	Sheep meat	27	0.86 ± 0.15	0.77461	0.00	3.05	
	Horse meat	63	0.95 ± 0.12	0.95107	0.00	3.11	
	Chicken	61	1.21 ± 0.17	1.32739	0.00	5.49	
	Pork	29	1.13 ± 0.23	1.25482	0.00	4.29	
Streptomycin	Beef meat	171	28.84 ± 4.82	63.05630	0.00	436.12	0.072
	Sheep meat	27	14.89 ± 4.21	21.88444	1.01	110.56	
	Horse meat	63	11.62 ± 1.99	15.77059	0.93	100.99	
	Chicken	61	37.75 ± 9.40	73.41574	0.00	492.00	
	Pork	29	23.83 ± 5.03	27.10374	1.47	111.19	
Tylosin	Beef meat	171	0.83 ± 0.09	1.15484	0.00	3.58	0.000
	Sheep meat	27	0.04 ± 0.03	0.14147	0.00	0.54	
	Horse meat	63	0.11 ± 0.07	0.52131	0.00	2.90	
	Chicken	61	2.68 ± 0.78	6.08781	0.00	44.36	
	Pork	29	0.58 ± 0.17	0.88966	0.00	2.42	
Tetracycline	Beef meat	171	7.13 ± 0.67	8.79212	0.00	52.00	0.005
	Sheep meat	27	4.23 ± 0.48	2.47002	0.00	8.89	
	Horse meat	63	3.63 ± 0.36	2.83351	0.00	9.79	
	Chicken	61	5.61 ± 0.50	3.89461	0.00	13.33	
	Pork	29	6.44 ± 0.88	4.75347	0.00	16.11	

*Mean ± standard error, N = Number of samples, min = Minimum, max = Maximum, δ = Standard deviation

- Quinolones: Concentrations ranged from 0.80 ppb in horse meat to 6.53 ppb in chicken, with a maximum of 91.97 ppb in chicken, suggesting failure in drug withdrawal practices (p = 0.000).
- CEFT: Most frequently detected in beef (2.82 ppb) and chicken (3.62 ppb), lowest in sheep (0.86 ppb), with significant interspecies variation (p = 0.000).
- Thiamphenicol: Concentrations were relatively uniform, highest in beef (1.58 ppb) and lowest in sheep (0.86 ppb), with statistically significant variation (p = 0.003).
- Streptomycin: Displayed wide variability, ranging from 11.62 ppb in horse to 37.75 ppb in chicken, with extreme chicken values reaching 492.00 ppb; differences were not statistically significant (p = 0.072).
- Tylosin: Ranged from 0.04 ppb in sheep to 2.68 ppb in chicken, with maximum values up to 44.36 ppb (p = 0.000).
- Tetracyclines: Highest in beef (7.13 ppb) and pork (6.44 ppb), lowest in horse and sheep, with significant differences (p = 0.005).

### Overall trends and public health concerns

Chicken meat consistently showed the highest antibiotic concentrations across multiple groups, reflecting the intensive and prophylactic use of antibiotics in poultry farming. Beef and pork exhibited moderate levels, while lamb and horse meat had relatively lower residues. Importantly, the detection of zero values in several samples indicates that safe production is possible under stringent veterinary controls. However, the presence of extremely high antibiotic levels in individual samples, particularly chicken, poses a serious public health concern. These findings emphasize the urgent need for stricter veterinary drug regulations, enforcement of withdrawal periods, and continuous surveillance of antimicrobial residues in food products.

## DISCUSSION

### Variation in antibiotic residues across meat and feed

The study by Silbergeld *et al*. [20] identified substantial variations in residual antibiotic levels across different meat types (beef, chicken, and sheep) and feed categories, consistent with recent scientific findings. These results highlight the urgent issue of unregulated antimicrobial use in livestock production and its potential consequences for both human health and the environment.

### Species-specific accumulation of residues

The highest antibiotic concentrations were observed in beef and mutton, possibly reflecting the extended fattening period typical for cattle and sheep [21]. For instance, sulfadimethoxine levels in beef (5.42 ± 0.19 ppb) were nearly twice those detected in chicken (3.71 ± 0.32 ppb). This suggests that longer production cycles in cattle may lead to greater drug accumulation, underscoring the importance of stricter adherence to withdrawal periods before slaughter.

### Antibiotic-specific observations

Elevated CEFT concentrations in chicken and beef were likely associated with its widespread use for treating respiratory diseases in livestock [22]. Streptomycin exhibited extreme variability, particularly in chicken samples (up to 492 ppb), which may be attributed to its dual application for therapeutic and prophylactic purposes [23]. Such variability reflects inconsistent and potentially uncontrolled dosing practices on farms. Alarmingly, some beef samples contained excessively high antibiotic levels, for example, dapsone (285.14 ppb), far exceeding the maximum permissible limits set by EAEU TR 051/2021. These findings strongly indicate a failure to comply with drug withdrawal requirements before slaughter, warranting urgent regulatory intervention [24].

### Feed as a source of contamination

The elevated levels of streptomycin in succulent feeds (86.43 ± 52.03 ppb) and quinolones in concentrated feeds (20.14 ± 13.55 ppb) highlight the need to monitor the entire feed-animal-product continuum [25]. The detection of these residues in feed suggests that contaminated feed may serve as a critical source of antibiotic accumulation in animal tissues, emphasizing the importance of feed regulation.

### Interspecies differences and regulatory implications

Statistically significant interspecies differences (p < 0.05) were identified for 11 of the 15 antibiotics tested (73%), providing strong evidence for the necessity of species-specific residue standards [26]. These variations highlight that veterinary practices, drug metabolism, and rearing conditions differ substantially among animal species. In addition, stricter regulations and inspections are particularly needed for imported feed varieties, where elevated antibiotic residues have been reported. Further regional studies across Kazakhstan are essential, especially since existing data remain limited for certain meat types such as horse and pork [16].

### Potential alternatives to antibiotic use

To address these challenges, alternative strategies such as probiotics and phytogenic feed additives offer promising solutions [27]. For instance, incorporating organic acids into feed has been shown to reduce antimicrobial use by 40%–60%. Variations in residue levels across meat types may also reflect differences in drug pharmacokinetics and animal husbandry practices. Transitioning toward these alternatives could help reduce dependency on antibiotics while improving livestock health and ensuring food safety.

## CONCLUSION

This nationwide surveillance study revealed significant variations in residual antibiotic levels in both meat and livestock feed samples collected from 14 regions of Kazakhstan. The findings demonstrated that succulent feeds contained the highest contamination, notably streptomycin (86.43 ppb) and quinolones (35.56 ppb), while coarse and concentrated feeds also exceeded detection thresholds, albeit at lower levels. Among meat products, chicken consistently exhibited the highest antibiotic residues, including quinolones (up to 91.97 ppb) and streptomycin (up to 492.00 ppb), followed by beef with alarmingly high concentrations of sulfadimethoxine (18.26 ppb) and dapsone (285.14 ppb). Statistically significant interspecies differences were identified for most antibiotic groups, highlighting the influence of species-specific veterinary practices and drug metabolism.

From a practical standpoint, these results underscore the urgent need for stricter veterinary regulation, routine monitoring, and enforcement of drug withdrawal periods before slaughter. The findings also suggest that feed contamination plays a critical role in residue accumulation, supporting the case for comprehensive regulation of both domestic and imported feed. Moreover, the study provides evidence for policymakers to strengthen Kazakhstan’s food safety frameworks in alignment with EAEU standards. The adoption of sustainable alternatives such as probiotics, phytogenic additives, and organic farming practices offers promising pathways to reduce antimicrobial dependence without compromising productivity.

A key strength of this study lies in its nationwide coverage and large sample size (1,026 meat and 150 feed samples), which provides a comprehensive overview of antimicrobial residue distribution across livestock sectors. The use of a validated biochip-based detection method ensured reliable sensitivity and specificity. However, several limitations should be acknowledged. The study did not evaluate seasonal variations, farm-level usage practices, or the direct link between residue levels and AMR in microbial isolates. In addition, certain livestock types (e.g., horse meat and pork) were underrepresented compared to beef and poultry, warranting broader sampling in future research.

Future studies should focus on longitudinal monitoring, farm-to-fork tracking of antibiotic use, and molecular assessment of resistant bacterial strains to better understand the public health implications of residues. Building a national database of antimicrobial usage and residue trends will be essential for guiding targeted interventions and strengthening compliance with international food safety regulations.

In conclusion, this study provides the first comprehensive evidence of widespread antibiotic residues in meat and feed across Kazakhstan, highlighting a critical food safety and public health challenge. The results call for immediate policy action, including stricter regulation, enhanced laboratory surveillance, and farmer education programs. By adopting sustainable livestock management practices and aligning with global antimicrobial stewardship initiatives, Kazakhstan can mitigate the risks of AMR, ensure consumer safety, and support the transition toward safe, sustainable, and resilient food production systems.

## AUTHORS’ CONTRIBUTIONS

AkZh: Conceptualization. AZh, UR, SZ, SK, and AO: Methodology, conducted experiments, and data collection. SG: Statistical analyses. AZh: Drafted and revised manuscript. All authors have read and approved the final version of the manuscript.

## APPENDIX A

**Appendix A:** Residual amount of antibiotics in meat detected using Ultra Antimicrobial Array I.

Antibiotics	Type of meat	n	Average, ppb	δ	min	max	p-value
Sulfadiazine	Beefmeat	190	2.08 ± 0.16	2.22	0.00	8.11	0.000
	Horsemeat	55	2.75 ± 0.30	2.19	0.00	5.34	
	Chicken	50	0.51 ± 0.18	1.24	0.00	4.17	
	Sheepmeat	26	2.71 ± 0.43	2.21	0.00	5.17	
	Pork	32	0.29 ± 0.20	1.13	0.00	4.74	
Sulfadimethoxine	Beefmeat	190	4.25 ± 0.22	3.01	0	14	0.011
	Horsemeat	55	4.27 ± 0.45	3.36	0	9	
	Chicken	50	3.67 ± 0.36	2.54	0	10	
	Sheepmeat	26	4.32 ± 0.68	3.47	0	8	
	Pork	32	2.27 ± 0.45	2.55	0	8	
Sulfaquinoxalin	Beefmeat	190	1.02 ± 0.07	1.00	0.00	4.07	0.000
	Horsemeat	55	1.28 ± 0.14	1.03	0.00	2.71	
	Chicken	50	0.46 ± 0.09	0.60	0.00	2.12	
	Sheepmeat	26	1.27 ± 0.21	1.05	0.00	2.73	
	Pork	32	0.16 ± 0.11	0.60	0.00	2.45	
Sulfamethazine	Beefmeat	190	1.24 ± 0.09	1.30	0.00	4.91	0.000
	Horsemeat	55	1.57 ± 0.17	1.24	0.00	3.05	
	Chicken	50	0.42 ± 0.11	0.76	0.00	2.78	
	Sheepmeat	26	1.58 ± 0.25	1.25	0.00	2.78	
	Pork	32	0.30 ± 0.12	0.67	0.00	2.88	
Sulfamethoxazole	Beefmeat	190	1.92 ± 0.22	3.01	0.00	35.00	0.000
	Horsemeat	55	2.27 ± 0.24	1.81	0.00	4.36	
	Chicken	50	0.61 ± 0.15	1.05	0.00	3.52	
	Sheepmeat	26	2.19 ± 0.35	1.78	0.00	4.20	
	Pork	32	0.23 ± 0.16	0.89	0.00	3.72	
Sulfathiazole	Beefmeat	190	1.76 ± 0.13	1.77	0.00	6.65	0.000
	Horsemeat	55	2.19 ± 0.24	1.75	0.00	4.13	
	Chicken	50	0.63 ± 0.14	1.02	0.00	3.93	
	Sheepmeat	26	2.20 ± 0.36	1.82	0.00	4.52	
	Pork	32	0.27 ± 0.18	0.99	0.00	4.13	
Sulfizoxazole	Beefmeat	190	1.80 ± 0.14	1.90	0.00	7.23	0.000
	Horsemeat	55	2.48 ± 0.27	1.99	0.00	5.21	
	Chicken	50	0.44 ± 0.15	1.05	0.00	3.89	
	Sheepmeat	26	2.45 ± 0.40	2.05	0.00	5.24	
	Pork	32	0.30 ± 0.21	1.16	0.00	4.79	
Sulfapyridine	Beefmeat	190	2.12 ± 0.13	1.80	0.00	7.19	0.000
	Horsemeat	55	2.37 ± 0.26	1.94	0.00	5.59	
	Chicken	50	1.10 ± 0.19	1.32	0.00	4.15	
	Sheepmeat	26	2.38 ± 0.34	1.73	0.00	4.35	
	Pork	32	1.26 ± 0.24	1.37	0.00	5.32	
Sulfamerazine	Beefmeat	190	2.13 ± 0.12	1.69	0.00	5.19	0.000
	Horsemeat	55	2.53 ± 0.27	2.01	0.00	4.56	
	Chicken	50	1.30 ± 0.15	1.08	0.00	4.00	
	Sheepmeat	26	2.46 ± 0.35	1.79	0.00	4,31	
	Pork	32	0.74 ± 0.18	1.03	0.00	4.38	
Sulfamomethoxin	Beefmeat	190	18.26 ± 5.89	81.18	0.00	1125.00	0.679
	Horsemeat	55	12.14 ± 1.29	9.57	0.00	24.50	
	Chicken	50	7.63 ± 0.88	6.20	0.03	23.23	
	Sheepmeat	26	13.75 ± 1.90	9.67	0.09	25.39	
	Pork	32	4.82 ± 0.96	5.40	0.00	25.30	
Sulfamethoxypyridazine	Beefmeat	190	2.71 ± 0.17	2.29	0.00	8.83	0.000
	Horsemeat	55	3.27 ± 0.36	2.63	0.00	6.89	
	Chicken	50	1.35 ± 0.24	1.67	0.00	4.90	
	Sheepmeat	26	3.13 ± 0.46	2.36	0.00	6.02	
	Pork	32	1.55 ± 0.24	1.36	0.00	5.70	
Sulfachloropyridazine	Beefmeat	190	2.23 ± 0.16	2.25	0.00	8.15	0.000
	Horsemeat	55	2.93 ± 0.32	2.36	0.00	6.38	
	Chicken	50	0.75 ± 0.17	1.19	0.00	4.28	
	Sheepmeat	26	2.69 ± 0.43	2.19	0.00	5.18	
	Pork	32	0.53 ± 0.19	1.06	0.00	4.55	
Dapsone	Beefmeat	190	9.04 ± 2.01	27.77	0.00	285.14	0.502
	Horsemeat	55	4.86 ± 0.77	5.72	0.00	26.42	
	Chicken	50	6.16 ± 1.22	8.61	0.00	43.42	
	Sheepmeat	26	4.48 ± 0.86	4.36	0.00	14.92	
	Pork	32	4.13 ± 1.80	10.16	0.00	57.24	
Sulfadoxine	Beefmeat	190	2.65 ± 0.19	2.66	0.00	9.69	0.000
	Horsemeat	55	3.31 ± 0.35	2.63	0.00	6.04	
	Chicken	50	0.91 ± 0.23	1.64	0.00	5.72	
	Sheepmeat	26	3.35 ± 0.53	2.72	0.00	6.20	
	Pork	32	0.36 ± 0.25	1.40	0.00	5.74	
Trimethoprim	Beefmeat	190	0.05 ± 0.01	0.15	0.00	0.87	0.169
	Horsemeat	55	0.06 ± 0.04	0.30	0.00	2.06	
	Chicken	50	0.01 ± 0.01	0.06	0.00	0.38	
	Sheepmeat	26	0.11 ± 0.05	0.24	0.00	0.77	
	Pork	32	0.02 ± 0.02	0.11	0.00	0.60	

*Mean ± standard error, N = Number of samples, min = Minimum, max = Maximum, δ = Standard deviation
